# LC-ESI-MS/MS Alkaloidal Profiling and Biological Investigation of *Cleistocactus winteri* Stems

**DOI:** 10.22037/ijpr.2020.1101080

**Published:** 2020

**Authors:** Suzan Adib Mina, Farouk Rasmy Melek, Rania Mohamed Adeeb, Eman Gaber Haggag

**Affiliations:** a *Department of Pharmacognosy, Faculty of Pharmacy, Helwan University, 1179, Cairo, Egypt.*; b *National Research Centre, Department of Chemistry of Natural Compounds, Dokki, 12622, Giza, Egypt.*

**Keywords:** Alkaloids, Cactaceae, Chromatography, Biology, Flavonoids

## Abstract

*Cleistocactus winteri* is a succulent plant of the *Cactaceae * family, commonly known as the golden rat tail. Many members of the *Cactaceae *family are the focus of chemical and biological studies as they contain bioactive compounds, well known for their health-related properties. We aimed to investigate *Cleistocactus winteri* stems both phytochemically and biologically for the first time including three biological effects. For the evaluation of the anti-inflammatory activity, Nitric oxide (NO) inhibition on lipopolysaccharide (LPS) stimulated Raw murine microphages (RAW 264.7) cell model was used. The cytotoxic activity was evaluated against hepatocarcinoma (HepG2), breast adenocarcinoma (MCF-7), colorectal carcinoma (HCT-116), and colon adenocarcinoma (CACO 2) human cancer cell lines using MTT (3-[4,5-dimethylthiazole-2-yl]-2,5-diphenyltetrazolium bromide) assay. The antioxidant activity was evaluated using 1,1-diphenyl-2-picrylhydrazyl (DPPH) assay. Seven alkaloids were tentatively identified based on Mass bank on line library in addition to the isolation and identification of four known flavonoids and a *β*-sitosterol 3-* O*-glucoside from the studied extract. The stem methanol extract showed a 6% inhibition of NO production (up to 48 μmol/L). Furthermore, the IC_50_ of the total alkaloid fraction against HepG2, MCF-7, and CACO-2 cell lines was equal to 26.53, 23.8, and 13.07 µg/mL, respectively, while that of the methanol extract against Hep-G2 and HCT-116 was 181 µg/mL and 357 µg/mL, respectively with no effect against MCF-7 cell line. The antioxidant effect of the extract was about one third (112 µg/mL) that of ascorbic acid.

## Introduction

*Cleistocactus winteri* is a succulent plant of the *Cactaceae* family which comprises approximately 130 genera and 1500 species (1, 2). It is commonly known as the golden rat tail. The stems are highly succulent and replace leaves in their photosynthetic function. Nowadays, the cactus fruits and cladodes are the focus of many studies as they contain bioactive compounds, well known for their health-related properties. Many members of the *Cactaceae* family have been proven to possess strong antioxidant properties (3-5) which make these plants take important role in protecting human health against degenerative diseases such as cancer (both *in-vivo* and *in-vitro* studies) (6-8), diabetes (9), and hypertension (10). Previous reports on the medicinal properties of family *Cactaceae* include also other biological effects such as anti-inflammatory (4), antiulcer (11), and hepato-protective activities (12). Among the 130 genera belonging to family *Cactaceae*, the genera *Opuntia, Pereskia, Eulychnia,* *Echinocereus*, *Echinopsis,* and *Stenocereus *were the most studied for their flavonoids (13), sterols and triterpene content (14, 15) while the others were mainly investigated for their alkaloidal content *e.g.* *Terbinicarpus* (16)*, Gymnocalcycuim *(17)*, Neobuxbaumia *(18)*, Desmoduim *(19),* Trichocereus, and Coryphantha *(20)*. *No previous phytochemical or biological studies were reported on genus *Cleistocactus* which was encouraging to carry out the evaluation of the anti-inflammatory and antioxidant activities of the 80% methanol extract of *Cleistocactus winteri (C. winteri) *stem using NO inhibition method on LPS-stimulated RAW 264.7 cells model and DPPH radical scavenging assay, respectively. The cytotoxic effect against four types of the human tumor cell lines, HepG2, MCF-7, HCT-116, and CACO 2 was evaluated for both the 80% methanol extract of the stems and the crude alkaloid fraction was derived from this extract. LC/ESI-MS/MS analysis was adopted for the investigation of the crude alkaloid fraction leading to the tentative identification of seven alkaloids belonging to phenylethylamines and tryptamines classes. Four known flavonoids, kampferol 3-*O-β-*D-glucopyranoside **1**, apigenin 8-*C*-*β***-**D-glucopyranoside **2,** quercitin 3-*O-β-*D-glucopyranoside **3a**, quercitin 3-*O-β-*D-galactopyranoside **3b**, were isolated and identified in addition to a beta sitosterol 3-*O-β-*D-glucopyranoside **4** based on the previous reports on the separation of different classes of flavonoids from *Cactaceae* family, including flavonols and dihydroflavonols and their glycosides (14, 21 and 22). It is worth mentioning that genus *Cleistocactus* had no previous neither about their biological activity nor about their phytochemical content. 

## Experimental

*Plant Material*

 The stems of *C. winteri* were collected from the International Cactus Farm, El Qanater El-Khairya- Qualiobya governorate, Egypt in May 2013. Plant identification was confirmed by Dr. Therese Labib, senior specialist for plant taxonomy in El Orman Garden, Giza, Egypt. A Voucher Specimen No. CW-38 was deposited in the herbarium of Pharmacognosy Department, Faculty of Pharmacy, Helwan University.

*LC/MS analysis*

 LC/MS analysis was performed on triple stage quadruple mass spectrometer, TSQ Quantum Access MAX, Thermo Scientific, New York, USA, equipped with electrospray ionization (ESI) operated in the positive ionization mode. Source temperature 150 °C, cone voltage 60 ev, capillary voltage 3 kv, desolvation temperature 440 °C, cone gas flow 50 L/h, and desolvation gas flow 900 L/h. Mass spectra were detected in the ESI positive ion mode between m/z 100–1000. Chromatography was carried on Accela U-HPLC system which was composed of Accela 1250 quaternary pump and Accela open auto sampler, New York, USA (operated at 25 ^o^C). The type of the column is Hypersil Gold column (C-18 bonded ultrapure silica based column) 50 mm × 2.0 mm (1.9 μm), Thermo scientific, New York, USA. Isocratic elution using fresh prepared Acetonitrile (A) was: 0.2% formic acid aqueous solution (B) (90:10, v:v) at room temperature. Flow rate (250 μL/min). X-caliber software version 2.2 was used to control all parameters of UPLC and MS and analysis of the obtained data. 

*Extraction of alkaloids*

 The air dried powder of *C. winteri* stems (1 kg) was extracted with 80% aqueous methanol (4 × 3 L) at 55 ^o^C. The dried methanol residue (60 g) was defatted with petroleum ether (4 × 0.5 L) to yield 45 g of defatted extract residue. The residue was shaken with 500 mL 10% HCl and left to stand for 24 h. After filtration the solution was extracted with chloroform three times. The chloroform layer was separated and ammonium hydroxide was added to the aqueous layer till complete neutralization. Extraction of the free alkaloids was done with 3 × 500 mL chloroform and the collected chloroform layer was washed with distilled water to remove the excess alkalinity. The obtained fraction was dried under reduced pressure to yield 95 mg of crude alkaloid fraction and the spots were monitored on TLC plate using solvent system *n*-butanol: acetic acid: water (40:10:10), they were visualized by dragendorff spray reagent. The alkaloid fraction was then analyzed by LC/ ESI-MS/MS. 

*Alkaloids identification*

 The crude alkaloid fraction was analyzed by LC/ESI-MS/MS using the positive ionization mode and the identification of the alkaloid components were based on computer matching of their mass spectral fragmentation patterns with those stored in Mass bank on line library (Mass Bank Version 2.4, supported by Institute for Bioinformatics Research and Development, Japan Science and Technology Agency for 2006-2010) and (National Institute of Standards and Technology (NIST) spectral search program for the NIST/EPA/NIH mass spectral library version 2.0) in addition to the comparison to literature data. 

*Extraction and isolation of compounds (1-4)*

 Another batch of dried powdered *C. winteri *(1.8 kg) was extracted three times with 80% methanol (3 × 3 L). After solvent evaporation and defatting with petroleum ether, the obtained brown residue (80 g) was dissolved in distilled water (0.002% w/v) then passed through a porous polymer gel Mitsubishi Diaion HP-20 column. Elution was carried out using distilled water, 25%, 50%, 75% MeOH and finally with 100% methanol. The 50% MeOH fraction (12 g) was chromatographed on polyamide column and eluted with 100% H_2_O and gradual decrease of polarity by addition of MeOH up to 100% MeOH. Ten collective fractions (I - X) were obtained based on TLC monitoring using CHCl_3_-MeOH-H_2_O (6:3:0.5). Visualization of spots was carried out by UV lamp and spraying with FeCl_3_. Fractions II (920 mg) and IV (700 mg) were repeatedly chromatographed separately on PC using solvent system CH_3_COOH-H_2_O (15:85) to afford after purification on Sephadex LH-20 column, compounds **1** (12 mg) and **2** (19 mg) from fraction II and compound **3a** and **3b** (22 mg) from fraction IV as an inseparable mixture. Fraction I (3 g) was subjected to silica gel VLC. Elution started with CH_2_Cl_2_ and the polarity was increased by addition of MeOH. Two percent MeOH fraction (800 mg) was applied on Sephadex LH 20 eluted with H_2_O then MeOH.The fraction eluted with 100% MeOH (100 mg) showed a major purple spot after spraying with 20% H_2_SO_4_ and heating at 150 ^o^C. The material from this fraction was further purified on PTLC using CH_2_Cl_2_: MeOH (9:1) to afford compound **4** (36 mg).

*Biological Material*

 Raw murine macrophage (RAW 264.7), human hepatocarcinoma cell line (HepG2), human breast adenocarcinoma cell line (MCF-7), human colorectal carcinoma cells (HCT-116) and human colon adenocarcinoma cell line (CACO-2) were purchased from the American Type Culture collections, Cambrex, BioScience (Copenhagen, Denmark). L-glutamine, penicillin G sodium, streptomycin sulphate, amphotericin B, MTT, isopropanol, LPS, dexamethasone, DPPH, ascorbic acid and DMSO were purchased from Sigma/Aldrich, (St. Louis, MO USA). The study followed principles in the Declaration of Helsinki. 

*Anti-inflammatory activity (NO inhibition method)*

 According to Green *et al.* (1982) raw murine macrophages (RAW 264.7) were seeded in 96-well plates at 0.5 × 10^5 ^cells/well for 2 h in RPMI-1645 without phenol red (23). The cells were stimulated with LPS with final concentrations of 100 µg/mL. Stimulated cells after two extra hours were either treated with 100 µg/mL (safe dose) of the test samples or dexamethasone (50 µg/mL), as a potent anti-inflammatory, left with the LPS alone, or left untreated at all as a negative control. After total 24 h time interval, the supernatants were removed and assessed for NO*. *In each well of a flat bottom 96-well- microplate, 40 µL freshly prepared Griess reagent was mixed with 40 µL cell supernatant of different concentrations of sodium nitrite (12.5, 20, 50, 100 µg/mL). The plate, incubated for 10 min in the dark and the absorbance of the mixture was determined at 540 nm using the microplate ELISA reader. A standard curve relating NO concentration in µM/L to the absorbance is constructed, from which the NO level in the cell supernatant is computed by interpolation. The NO level of each of the tested cell supernatant was expressed as NO level of the tested cell supernatant ×100/NO level of the control (1). All experiments were repeated three times and the data was represented as (Mean ± SD).

*Cytotoxic activity (MTT assay)*

Four human tumor cell lines, HepG2, MCF-7, CACO-2 and HCT-116 were used in this study. The cytotoxic activity was tested by MTT assay according to Mossman (1983) (24). The cells (0.5 × 10^5^ cells/well) were cultured in RPMI 1640 supplemented with 10% heat inactivated fetal bovine serum (FBS) and 1% penicillin/streptomycin and maintained at 37 °C in an atmosphere of 5% CO2. The cells were placed in a flat bottom TC 96-well micro plate, and treated with 20 µL of different concentrations of the test samples (12.5, 25, 50, 100 μg/mL) for 24 h. After incubation, the media were removed and 40 µL MTT solution per well were added and incubated for an additional 4 h. MTT crystals were solubilized by adding 180 µL of acidified isopropanol per well and each plate was shacked at room temperature followed by photometric determination of the absorbance at 570 nm using micro plate ELISA reader. Four times repeats were performed for each concentration and the average was calculated. The data were expressed as the percentage of relative viability compared with the untreated cells and with the vehicle control, with cytotoxicity indicated by <100% relative viability. The percentage of relative viability was calculated using the following Equation:

[Absorbance of treated cells/Absorbance of control cells)] × 100

The concentration of extracts required to kill 50% of cell population (IC_50_) was determined from the data generated by plotting a dose-response curve. IC_50_ value was calculated by nonlinear regression of log (concentration) versus% survival.

*Antioxidant activity (DPPH assay)*

 The ability of the extract to scavenge free radicals was assayed according to Brand-williams *et al.* (1995) (25). In a flat bottom 96-well microplate, a total test volume of 200 µL was used. In each well, 20 µL of different concentrations (12.5, 25, 50, 100 µg/mL final concentration) of the test samples were mixed with 180 µL of ethanol DPPH and incubated for 30 min at 37 ºC. Triplicate wells were prepared for each concentration and the average was calculated. Photometric determination of absorbance at 520 nm was performed by microplate ELISA reader. The half maximal scavenging capacity (SC_50_) values for each test sample and ascorbic acid used as positive control was estimated via dose-response curve. SC_50_ of each sample was calculated using the curve equation. 

## Results and Discussion

**Figure 1:**
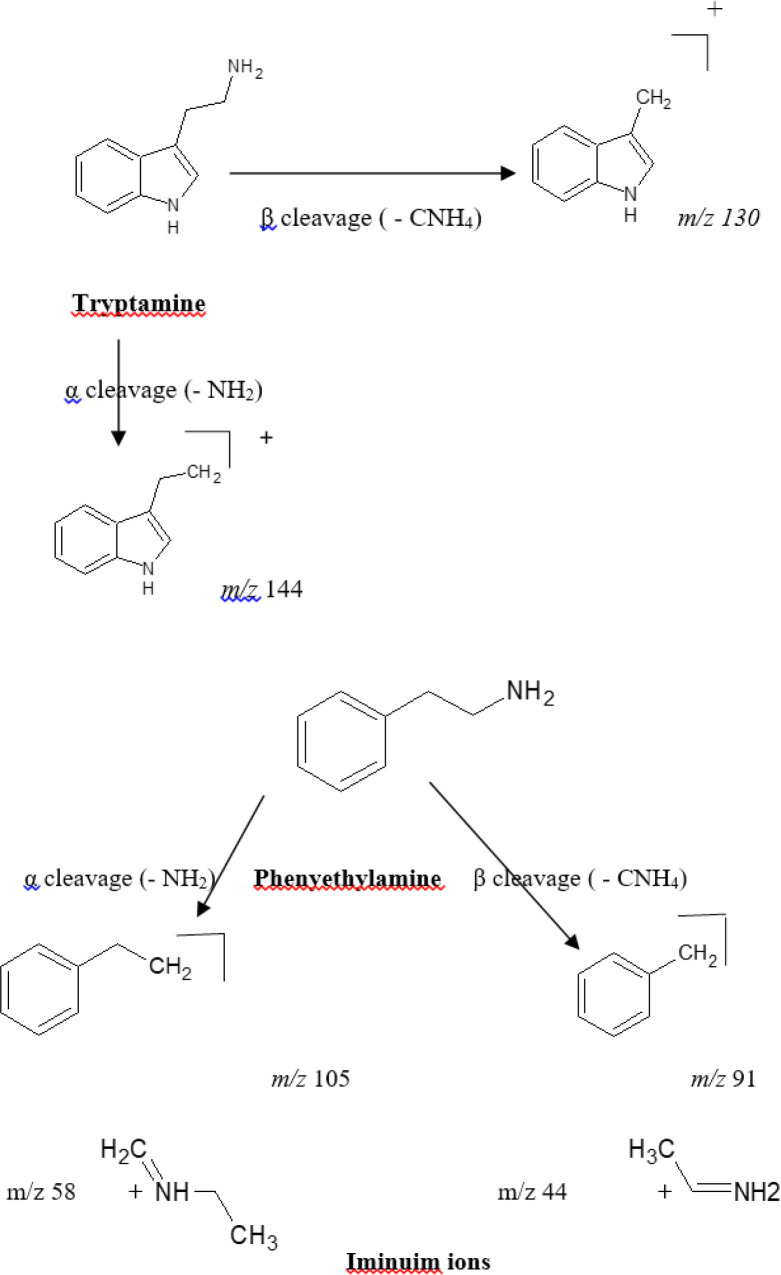
Tryptamine and phenylethylamine fragmentation pattern

The mass spectra of the alkaloids were investigated to determine diagnostic ions depending on the structural characteristics of alkaloids in the *Cactaceae* family. Upon scanning of the crude alkaloid fraction, seven alkaloids were tentatively identified according to their fragmentation pattern to be based on two backbone structures, phenylethylamines and tryptamines (indole alkaloids) (Table 1, Figure 1). Four of the seven tentatively identified alkaloids, were related to the parent compound *β*-phenylethylamine. These compounds showed a typical fragmentation pattern with marker characteristic mass fragments appearing at *m/z* 91 corresponding to *β*-cleavage and the fragment ion appearing at *m/z* 105/106 (for the cation and radical cation) corresponding to an α-cleavage of the side chain in the phenylethylamine structure as well as the fragment ion corresponding to the phenyl cation at* m/z *77 [C_6_H_5_]^ +^. Varied degree of N-substitution and oxygenation of the benzene ring and alkyl chain are commonly seen in the produced ion fragments. The four alkaloids were identified as phenyl ethyl amine [M+1, *m/z* 122], tyramine [M+1, *m/z* 138], Synephrine [M+1, *m/z* 168] and 3, 4 methylene dioxy-N-ethylamphetamine [M+1, *m/z* 208]. The remaining three alkaloid components demonstrated characteristic key fragmentation pattern for tryptamines showing the following series: *m/z* 144, 130, 103 and 77 indicating tryptamine derivatives (Figure 1) (26). The three alkaloids were identified as Melatonin [M+1, *m/z* 233], 5-hydroxytryptophan [M+1, *m/z* 221] and 5- methoxytryptamine [M+1, *m/z* 192]. The appearance of an ion fragment at *m/z* 77 in the seven tentatively identified alkaloids indicates the dissociation of the phenyl cation [C_6_H_5_]^+^ fragment. It is worth mentioning that the abundance of peaks representing different fragments varies with the condition of the analysis including the energy used for fragmentation, and the stability of the formed fragments. 

Five compounds were isolated from the stem aqueous methanol extract of *C. winteri *and they were identified as Kampferol 3-*O*-*β*-D-glucopyranoside (27), quercitin 3-*O*-*β*-*D*-galactopyranoside and quercitin 3-*O*-*β*-*D*-glucopyranoside) (28-30) and the flavones C-glycoside, apigenin 8-C*-β*-*D*-glucopyranoside (vitexin) (31, 32) together with the steroidal glycoside *β*-sitosterol 3-*O*-*β*-*D*-glucopyranoside (33). Identification of these compounds was based on UV and NMR analysis as well as by comparing the data with the reported literature values.

*Biological investigations*

*Antiinflammatory activity evaluation*

**Figure 2:**
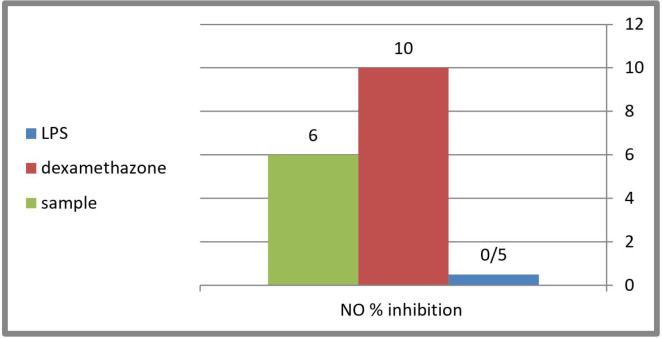
Percentage of NO inhibition of *C. winteri* extract compared to Dexamethasone

 Evaluation of the anti-inflammatory activity was done using (Nitric Oxide) NO inhibtion method. The 80% methanol extract of *C. winteri *stems showed a moderate anti-inflammatory effect with a NO production level up to 48 μmol/L compared to the potent anti-inflammatory dexamethasone used as positive control (46 μmol/L) (Figure 2). Nitric oxide, being a marker for inflammation, is induced by LPS (inflammagen lipopolysaccharide) through the expression of inducible nitric oxide synthase (INOS) (a pro-inflammatory mediators). Therefore, the examination of the ability of the extracts to suppress the release of pro-inflammatory mediators in an LPS-stimulated RAW 264.7 cells model, could indicate their ability to be used for the treatment or prevention of inflammation and also gives an idea about their mechanism of action. The results indicated that the inflammagen (LPS 100 μg/mL) induced nitric oxide production up to 1.2 folds of the control, while the potent anti-inflammatory dexamethasone (50 μg/mL) inhibited nitric oxide production up to 46 μmol/L compared to 51 μmol/L of that of the LPS with a 10% inhibition level. The 80% methanol extract of *C. winteri *stems possess a moderate anti-inflammatory effect with an inhibition percentage equal to 6% compared to the potent anti-inflammatory dexamethasone used as a positive control (Figure 2). 

*Cytotoxic activity evaluation*

**Figure 3:**
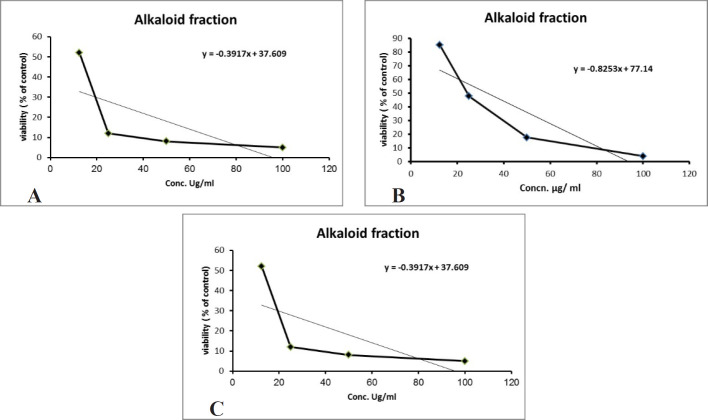
(A) Cytotoxic effect of the alkaloid fraction against Hep-G2 (r^2 ^= 0.77), (B) MCF-7 (r^2 ^= 0.78) and (C) CACO-2 (r^2 ^= 0.46) cells. All cytotoxic effects were done using MTT assay (n = 4), data expressed as the mean value of cell viability (% of control) ± SD

**Figure 4:**
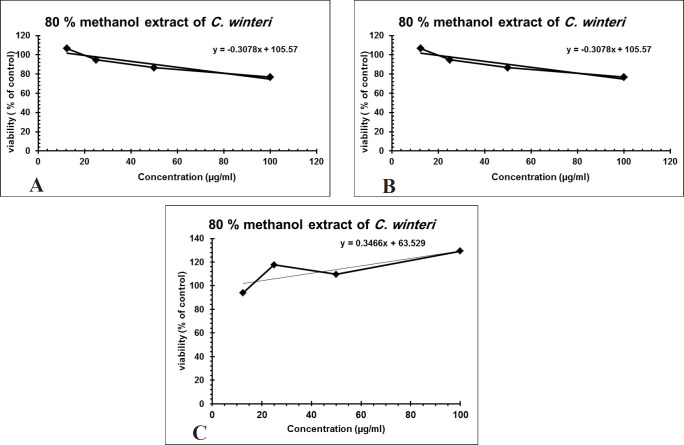
(A) Cytotoxic activity of *C. winteri* methanolic extract against Hep-G2 (r^2 ^= 0.908), (B) HCT-116 (r^2 ^= 0.446), and (C) MCF-7 (r^2 ^= 0.65), All cytotoxic effects were done using MTT assay (n = 4), data expressed as the mean value of cell viability (% of control) ± SD

 Over 50% of the drugs used in clinical trials for anticancer are of natural origin or related to them (34). The *in-vitro* evaluation of the cytotoxic activity of the crude alkaloid fraction against three human cancer cell lines [(HepG2), (MCF-7) and (CACO-2)] using MTT assay showed remarkable cytotoxic effect against the three tested cell lines with an IC_50_ equal to 26.53 µg/mL, 23.8 µg/mL, and 13.07 µg/mL, respectively, having nearly equivalent activity to that of Doxorubicin was used as a positive control (Figures 3A-3C). The cytotoxic activity of the crude alkaloid fraction is seen from the rapid strong reduction of the viability of the used cell lines specially HPG2 and CACO-2 types where a slight increase in the used concentration (25 μg/mL) of this fraction causes an abrupt reduction in the % viability of the tested cells. While in the case of breast cancer cell line (MCF7), an inversely proportional relation was seen between the increase in the concentration of the alkaloid fraction and the percent viability of the tested cells indicating also a remarkable activity against this type of cancer cells. The strongest effect is seen against the CACO-2 type with the lowest IC_50_ (13.07 µg/mL) of the three tested types of cancer cell lines. These results are in conjunction with previous reports on established dose dependent growth inhibition of the cancer cells of other members of the *Cactaceae* family (35, 36). The effect of the aqueous methanol extract on the proliferation of Hep-G2, MCF7 and HCT-116 cells were studied after 24 h of incubation (Figure 4). The results showed weak activities against Hep-G2 and HCT-116 cell lines with IC_50_ equal to 181 µg/mL and 357 µg/mL, respectively. No effect was observed against MCF-7 cells (Figures 4A-4C). 

*The antioxidant activity evaluation*

 The antioxidant potency showed significant relationship with the total phenolic content of the tested plant extracts since phenolic compounds are good electron donors and could terminate the radical chain reaction by converting free radical to more stable products. For the evaluation of the antioxidant activity, using DPPH assay, the 80% stem methanol extract was found to possess antioxidant effect equal to about one third (112 µg/mL) that of ascorbic acid (40.3 µg/mL) used as positive control.

LPS (lipopolysaccharide) and the sample under investigation.

**Table 1:** Alkaloids identified from the stems of *C. winteri* and their fragmentation pattern

	Name	M+1	MW	Fragmentation (*m/z*) (Relative intensity, %)
1	Phenylethylamine	122	121	77 (74), 91 (12), 103 (42), 106 (99), 122 (18)
2	Tyramine	138	137	55 (28), 65 (38), 77 (99), 81 (32), 91 (30), 94 (8), 103 (10), 138(2)
3	Synephrine	168	167	77 (44), 79 (36), 106 (99), 107 (66), 115 (18), 117 (32), 120 (40), 148 (40), 164 (6)
4	Methoxy tryptamine	192	191	77 (8), 105 (80), 117 (16), 130 (46), 132 (34), 160 (99), 174 (10), 192 (2)
5	Methylene dioxy-N-ethyamphetamine	208	207	77 (28), 91 (48), 105 (99), 133 (86), 163 (46), 174 (22), 192 (8), 208 (4)
6	Hydroxytryptophane	221	220	65 (26), 77 (56), 105 (36), 107 (99), 131 (52), 146 (58), 188 (16), 221 (2)
7	Melatonin	233	232	43 (22), 77 (34), 90 (36), 117 (40), 130 (34), 144 (34), 160 (99), 174 (20), 233 (14)

MW = molecular weight.

## Conclusion

LC/ESI-MS/MS technique resulted to be an effective tool for the identification of alkaloids present in the stems of *C. winteri*. The selectivity of this technique allows the characterization of compounds not only by their pseudomolecular ions but also by their specific fragmentation patterns. The aqueous methanol extract of *C. winteri* showed moderate both anti-inflammatory and antioxidant effects. The alkaloidal content of *C. winteri* showed strong cytotoxic activity which still needs further investigation of the mechanism of action for better interpretation and understanding of the established activity. 
